# Feasibility of recording EEG in the ambulance using a portable, wireless EEG recording system

**DOI:** 10.1371/journal.pone.0327415

**Published:** 2025-07-01

**Authors:** Sampsa Lohi, Pekka Jäkälä, Jouni Kurola, Pasi Tuunanen, Susanna Westeren-Punnonen, Anu Muraja-Murro, Reetta Kälviäinen, Esa Mervaala

**Affiliations:** 1 Department of Clinical Neurophysiology, Kuopio University Hospital, Member of ERN EpiCARE, Kuopio, Finland; 2 Department of Neurology, Kuopio University Hospital, Kuopio, Finland; 3 Institute of Clinical Medicine, School of Medicine, Faculty of Health Sciences, University of Eastern Finland, Kuopio, Finland; 4 Centre for Prehospital Medicine, Kuopio University Hospital, Kuopio, Finland; 5 Kuopio Epilepsy Centre, Neurocenter, Kuopio University Hospital, Member of ERN EpiCARE, Kuopio, Finland; Aalto University School of Science and Technology: Aalto-yliopisto Insinooritieteiden korkeakoulu, FINLAND

## Abstract

**Objective:**

Triaging acute ischemic stroke patients is difficult in prehospital settings. We investigated if a quickly applicable and compact EEG recording system is usable for stroke patients in the ambulance.

**Methods:**

The EEG of 10 acute stroke patients from Kuopio University Hospital, Finland was recorded using a forehead EEG electrode set and compact EEG amplifier-recorder during their ambulance transfer to another healthcare facility. The recordings were transmitted wirelessly in real time to our server, and their quality and the interruptions and technical difficulties in the wireless data transfer were analysed.

**Results:**

In 9 of the 10 recordings, the signal quality was sufficient for interpreting at least half of the recording. The signal quality suffered from artefacts caused by the patients’ movements and the loosening of the electrode contacts. Only 50-Hz AC artefacts that affected the reference electrode were considered obtrusive and required digital filtering in two recordings.

**Conclusions:**

We demonstrated that adequate-quality EEG can be recorded in an ambulance using a quickly applicable and compact recording system and reliably transferred to a remote server for real-time review.

**Significance:**

This system can be used to measure EEG in acute indications in the prehospital setting.

## Introduction

Stroke is the third leading cause of mortality worldwide and the fourth most common cause of lost disability-adjusted life years [1]. In acute ischemic stroke, there is a limited window of opportunity to save a critically hypoperfused but still viable brain tissue through reperfusion, limiting the extent of the final infarct. The length of this window is determined mainly by individual factors, one of the most important of which is the collateral blood flow to the affected brain. In individuals with poor collateral blood flow, the hypoperfused brain progresses faster into infarcted brain tissue, whereas in individuals with good collaterals, this ‘penumbra’ stays viable for a longer time. In the clinical setting, a mismatch between the infarct volume and a critically hypoperfused brain indicates the amount of remediable tissue. Reperfusion may be attempted using a clot-dissolving infusion (intravenous thrombolysis), by mechanically removing the clot (intra-arterial thrombectomy) or a combination of both [2].

Approximately one-third of acute stroke patients present with a large-vessel occlusion (LVO). These patients have a higher risk of death or disability [3] and could benefit from direct transfer to a comprehensive stroke centre for mechanical clot removal (thrombectomy). A few clinical scales can have acceptable to good accuracy in detecting LVOs in prehospital settings; however, objective technical methods could still prove more reliable [4].

Surface or scalp EEG is a non-invasive method of assessing brain function. Electrodes are placed on the scalp at predetermined locations, symmetrically on both sides of the head, and their measured voltage fluctuations are compared with those of a reference electrode that is usually also placed somewhere on the scalp. The measured fluctuations mainly reflect the postsynaptic currents in the pyramidal cells in the cerebral cortex, which is especially susceptible to ischemia [5]. Increasing levels of ischemia result in more pronounced changes in the scalp EEG. In mild to moderate ischemia (15–35 mL/ 100 g/min), faster EEG activities (alpha and beta, or frequencies over 8 Hz) decrease. When the severity and duration of ischemia increase, a shift towards slow wave activity (delta, or frequencies below 4 Hz) is seen first, followed by attenuation and eventual disappearance of all EEG activity. These characteristic EEG changes may be evident as early as 20 s after the onset of ischemia. As such, EEG is capable of delivering continuous and timely information about changes in a patient’s brain function. EEG still remains the only means to reliably detect epileptic seizures, which are not an uncommon complication of acute stroke [6].

Several quantitative measures of cortical ischaemia from EEG have been investigated. Many of them calculate the ratios of power in the different frequency bands (beta, alpha, theta and delta) or the ratio of their combined power [5]. The brain symmetry index (BSI) shows asymmetries in the spectral power of the hemispheres [7] and it has been used to monitor stroke patients receiving intravenous thrombolysis [8]. An alternate approach computes a pairwise-derived BSI, which is also sensitive to focal EEG asymmetries [9].

A few articles have underlined the potential value of recording EEG in acute stroke patients [9–11], but several key factors limit the usability of EEG in emergencies. Most currently available commercial systems are unwieldy, slow and difficult to set up, and require expertise in both recording and interpreting the EEG [12]. Thus, we came up with the following list of requirements for an EEG recorder that can be used to evaluate acute stroke patients in emergencies: (1) compact and easy enough to set up as to require minimal to no training; (2) due to the time-critical nature of acute stroke treatment, can be set up in advance during ambulance transfer, thus preventing further delays once the patient has arrived at the emergency department; (3) can output the recording for remote viewing by a specialist in real time; and (4) does not cause notable interference in any of the standard diagnostic studies of acute stroke patients, especially in head computed tomography (CT) imaging.

Published research on registering prehospital EEG during emergency medical transport of stroke patients is scarce. We identified only two clinical trials on this subject with published results. Unlike our trial, both of these studies used dry electrodes to record the EEG.

The first of these, called the ‘EEG controlled triage in the ambulance for acute ischemic stroke’ (ELECTRA-STROKE), uses a cap of 8 dry electrodes manufactured by Eemagine Medical Imaging Solutions GmbH, Berlin, Germany [10,13]. This study of 311 suspected acute stroke patients demonstrated that prehospital EEG can be highly accurate in diagnosing anterior LVO (LVO-a) stroke. It reported that the combined weighted phase lag index and relative theta power was the best determinant of LVO-a stroke (sensitivity, 100% and specificity, 84%), followed by the theta–alpha ratio (sensitivity 75%, specificity 81%), demonstrating the value of recording EEG in suspected acute stroke patients. However, in about one-third of the patients in the study, poor data quality turned out to be a limiting factor. The authors concluded that further improving the quality of the recorded EEG is necessary.

The second, called ‘Evaluating the feasibility of prehospital point-of-care EEG: The prehospital implementation of rapid EEG (PHIRE) study’, uses a headband of 10 dry electrodes manufactured by Ceribell Inc, Sunnyvale, USA [14]. Their study of 34 patients with suspected seizure, stroke or altered mental status reported a higher rate of recordings with at least some channels free of artefacts (about 90%). However, only 71% of recordings had at least 5 minutes of artefact-free EEG on at least 6 channels, and only 47% of recordings had a high-quality connection in all electrodes.

These important studies [13,14] clearly warranted further evaluation of additional solutions to improve the technical quality of prehospital EEG. We conducted a feasibility study using a compact EEG amplifier-recorder and a quickly applicable forehead electrode set using wet EEG electrodes.

## Materials and methods

### EEG recording device

BrainStatus, a disposable forehead electrode set [15,16], has been tested for use to exclude nonconvulsive status epilepticus in acute patients with altered mental states in Kuopio University Hospital [17]. In this study of 100 acute patients, performance of the BrainStatus system was found comparable to a full-scalp clinical EEG in cases where there was no status epilepticus present. We wanted to investigate whether the same device could meet our criteria for use in acute stroke patients during their ambulance transfer. The BrainStatus system consists of (1) the disposable electrode set that is placed on the patient’s forehead and temples; (2) an amplifier-recorder and (3) an optional tablet computer interface. BrainStatus is produced by Bittium Biosignals Ltd. (Oulu, Finland) and is CE-marked for medical use.

The electrode set is screen-printed using silver ink and includes six frontal channels (Fp1, Fp2, Af7, Af8, F7 and F8), two zygomatic channels (Sp1 and Sp2) and two mastoid channels (T9 and T10) for EEG. In addition, it has an electro-oculography (EOG) channel below the right eye, two reference channels in the frontal midline and two ground channels above the eyes. The electrode contacts are coated with hydrogel and have adhesive pads around them to help both the electrode and the conducting hydrogel to stay in place. The electrode set connects to the amplifier via an adapter, which includes a channel for ECG and additional contacts for four EEG channels (that are not part of the default configuration). Thus, the default configuration with the electrode set and ECG consists of 12 active channels, with a total device maximum of 16 active channels.

The amplifier is compact and lightweight (see Table 1), with an internal battery whose charge lasts up to 24 h in its default configuration. It stores the recorded EEG in memory (on a separate SD card) and optionally connects to a tablet computer via Bluetooth.

**Table 1:** Technical specifications of the BrainStatus compact amplifier.

**Unipolar channels**	16 (10 active, 2 reference, 2 ground, 1 EOG, 1 ECG)
**Channel configuration**	Active: Fp1, Fp2, Af7, Af8, F7, F8, Sp1, Sp2, T9, T10 Reference: 2 electrodes (forehead) Ground: 2 electrodes (forehead)
**EOG**	Yes
**ECG**	Yes
**EEG sampling frequency**	250 Hz (4,000 Hz/ 16 channels)
**Data logger file format**	EDF (open format)
**Dimensions**	76 × 54 × 22 mm
**Weight**	80 g
**Operating time**	Up to 24 h with 12 channels

EOG, electrooculogram.

The tablet computer can display the signal data sent to it by connected devices and relay them to a remote server in real time, where it can be accessed by an authenticated user for an immediate review. It also stores the received data in its internal memory for backup.

**Fig 1:**
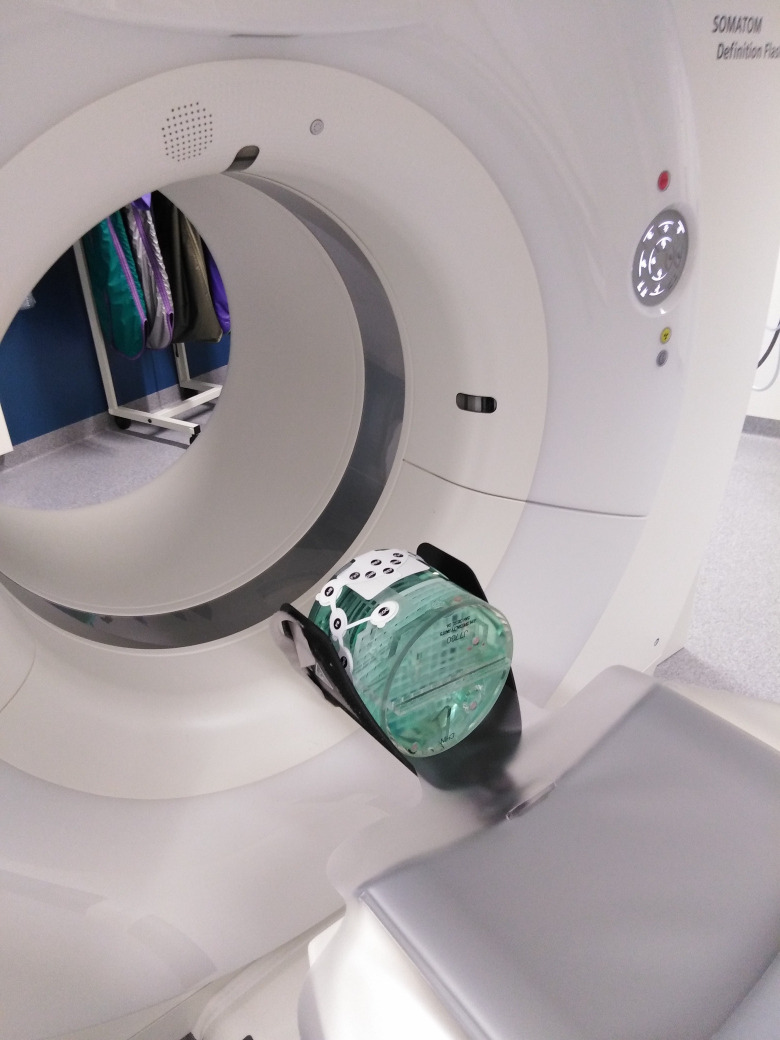
Testing for CT compatibility. Imaging phantom used to assess possible artefacts from the electrode in computed tomography images.

As part of this feasibility trial, we tested the electrode for CT compatibility using a so-called ‘imaging phantom,’ on which the forehead band was placed (Fig 1). Using an emergency head CT scanning protocol, a CT scan of this phantom was taken, and the resulting images were visually assessed by a medical physicist and a neuroradiologist. Both concluded that any interference caused by the forehead band on the phantom was negligible and would not affect the evaluation of head CT scans of acute stroke patients. Thus, the electrode band does not have to be removed during CT imaging.

### Remote server

Our configuration also included an on-premises server to which all the recorded EEG data were sent in real time during the ambulance transfer over an encrypted connection. Possible gaps in transferred data can be fixed by synchronising the remote server recording with the recording stored on the tablet computer once the patient has arrived at the hospital. Since our hospital district is scarcely populated and has long distances between stroke centres, we also wanted to assess the reliability of the wireless data transfer for possible real-time remote evaluation. Thus, the data that were saved on the remote server during the transfer were analysed as is using the BrainStatus software on desktop computers.

### Study population

During the recruitment period from October 20^th^ 2019 to August 19^th^ 2020 we recruited 10 adult patients, 6 of them male and 4 female, between the ages of 51 and 86 years who were being treated for acute stroke at the neurological and neurosurgical wards of Kuopio University Hospital in Finland and who required transfer by ambulance to another healthcare institution for further care. Their aetiologies included ischemic events (3) and haemorrhages (7). The recording was set up in advance at the patient ward and was continued until the ambulance reached its destination. The patients were instructed to remain calm and spend at least some time with their eyes closed during the transfer, however these periods were not evaluated separately from the rest of the recording. The signal quality, including artefacts and possible gaps in the data transfer, was evaluated. The patients gave their written informed consent to take part in this study, and the study protocol was approved by the Research Ethics Committee of the Hospital District of Northern Savo in Kuopio, Finland.

### In the ambulance

The monitoring tablet was set up to automatically connect to a WiFi network inside the ambulance and to transfer the data using a secure tunnel connection. We encouraged the emergency medical services (EMS) personnel in the ambulance to report any issues with the appliance immediately during the transfer by phone or by using a structured feedback form afterwards.

### Signal analysis

All 10 recordings were carried out successfully; no recording had to be excluded due to technical issues. In 2 of these recordings, no data loss occurred at all; and in 7 recordings, data loss occurred only during 1.5% of the total transit time at most. The remaining recording stood out with considerably longer interruptions—during 19.4% of the total transit time at most, with 2 of the interruptions lasting more than 6 min (Table 2).

**Table 2:** Patient transport and EEG data transfer interruption statistics.

Property	Mean	Range
Transport duration (hh:mm:ss)	1:42:00	0:41:35–3:23:04
Number of data transfer interruptions	8	0–22
Duration of single data transfer interruption in seconds	3.9	1–377
Total interruption time as percentage of transport time	2.71	0–19.41

The initial analysis was performed by SL. Specific questions were formulated to further assess the issues identified in the analysis. Two experienced clinical neurophysiologists from Kuopio Epilepsy Centre (AMM and SWP), both of whom had previous experience working with the BrainStatus, separately and blinded to the patients’ state evaluated all 10 EEG recordings to answer the following questions:

1. Does the recording contain interpretable EEG, EOG and ECG signals?
2. Is the technical quality consistent throughout the recording?
3. To what extent do movement artefacts (from the movements of the head and the eyes) affect the quality of the recording?
4. To what extent do artefacts from poor electrode–skin contact affect the quality of the recording?

Any significant issues not addressed by these questions were also reported.

## Results

All but one recording contained mostly (i.e., for more than half of the recording) interpretable signal data. One recording suffered from significant artefacts from both the patient’s movements and failing electrode contacts; and due to its poor quality, it was excluded from the rest of the analysis.

Most of the remaining recordings (7/9 according to AMM, and 8/9 according to SWP) had consistent quality throughout the recording. The one recording that both assessors agreed had inconsistent quality started to degrade after an hour of transit.

Most of the remaining recordings (8/9 according to both assessors) contained, at most, a moderate amount of movement artefacts. One recording had a notable number of eye movement artefacts.

Most of the remaining recordings (3/9 according to AMM, and 7/9 according to SWP) had some artefacts that were attributable to issues with the electrode–skin contact. The number of electrodes affected by these artefacts ranged from 1 to 4 out of 10. Most of these artefacts were 50-Hz AC artefacts, but loose-contact artefacts were also occasionally seen. The 50-Hz AC artefacts were considered especially obtrusive when they affected the reference electrodes and thus, were reflected on all the recorded channels.

**Fig 2:**
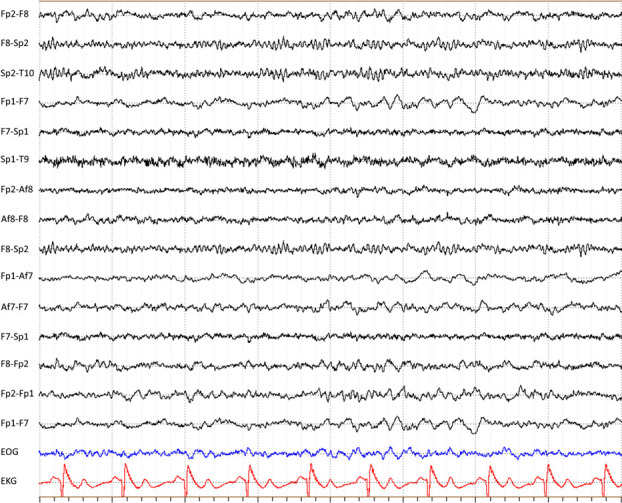
Sample 1 of an EEG recording during an ambulance transfer. Slowing is apparent in some of the left-side derivations (especially in Fp1-F7, Fp1-Af7 and Af7-F7). Fast artefact activity can be seen in the Sp2 electrode.

**Fig 3:**
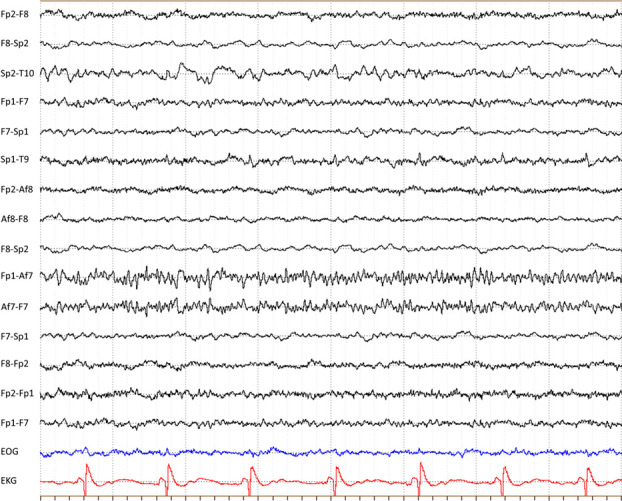
Sample 2 of another EEG recording during an ambulance transfer. Slowing and attenuation of faster frequencies is apparent in some of the right-side derivations (especially in F8-Sp2 and Sp2-T10). Fast artefact activity can be seen in the Af7 electrode.

Additional observations from the assessors included the presence of large-amplitude and slow baseline fluctuations in the frontopolar electrodes in most of the recordings, at least partially due to eye and eyelid movement artefacts. Some of the recordings (3/9 according to AMM, and 2/9 according to SWP) also contained intermittent artefact-like beta-frequency activities (Figs 2 and 3). Further analysis of these beta-frequency signals revealed that they varied greatly in frequency and amplitude and were unlikely to have been caused by any single, outside interference. A notch filter had to be used to analyse two of the remaining recordings that had 50-Hz AC artefacts in the reference electrode.

### Feedback from emergency medical services personnel

The only usability issue reported by the EMS personnel was the heating up of the monitoring tablet when kept inside a carrying case. This problem was noticed during the transfer of the first patient and was avoided after that by keeping the monitoring tablet outside the carrying case while the device was recording.

In 9 of the 10 patient transfers, the device was set up to use the ambulance’s own WiFi network. For one transfer, we tested a fallback mobile data connection (i.e., an optional SIM card for the monitoring tablet) and found that apart from a slightly longer delay when switching from the hospital’s WiFi network to the mobile data network, the data transfer performance during the transport was comparable to when the WiFi connection was used.

## Discussion

van Stigt et al. [13] have recently reported with a large patient population of 311 suspected stroke patients that prehospital EEG has high diagnostic accuracy. In this seminal study, however, the authors noted that EEG data suffered from insufficient quality thus preventing proper analysis in 32% of the patients. They suggested that future work should specifically address this issue and improve the technical quality of EEG data. In this feasibility study, we demonstrated that the Bittium BrainStatus EEG setup can be used to record good-quality EEG for long periods at a time in the ambulance. In 9 of the 10 cases that we examined, the technical quality of the acquired EEG signal was sufficient for clinical use. While the ELECTRA-STROKE study [13] used dry EEG electrodes, our feasibility study provides evidence that adhesive wet EEG electrodes may considerably improve technical quality of the recorded EEG data. In the two previously published studies EEG was recorded for relatively short periods of time (for a median of 151 seconds in the ELECTRA-STROKE study [13] and a median of 10.5 minutes in the PHIRE study [14]). The mean EEG recording time during patient transport in our study was 102 minutes, suggesting that even prolonged EEG recordings with sustained signal quality can be obtainable with this setup. Wireless transfer of the recorded signal to a remote server worked reliably in 9 of the 10 recordings.

In addition to the technical challenges regarding signal acquisition, the prehospital environment imposes additional requirements to the EEG recording device itself. The International Electrotechnical Commission (IEC) recommends a degree of ingress protection for electrical devices used by the EMS [18]. During the course of this trial, our device manufacturer developed a prototype EEG electrode that meets these requirements.

This study has a few limitations. We believe that our total of 10 trial recordings is sufficient for encountering and identifying any significant technical issues in this setup, but the number may be too low for robust statistical analysis. Although an EEG recording with the BrainStatus system can be started by a single person within minutes in a hospital environment [17], a moving ambulance can make electrode application more difficult. However, once at least part of the electrode has been placed, the adhesive pads around the contacts should hold the electrode in place and help in attaching the rest of the contacts.

A major advantage of the electrode’s position on the patient’s forehead is its minimisation of the artefacts caused by the movement of the patient’s head against the pillow during the transfer. However, this position of the electrode has a drawback as well—the EEG outside of the frontal and temporal regions is not recorded [17]. Clinically, LVOs in the anterior circulation (especially in the median carotid artery) usually cause wide-field slowing in the affected hemisphere [19] and should be picked up by the forehead electrode as notable interhemispheric asymmetry, while occlusions of the posterior circulation (i.e., the basilar artery) would likely be unnoticed. Our ultimate goal is to determine if EEG recorded with this system in the prehospital setting can be used to detect anterior LVOs for the purpose of directing these stroke patients to a centre capable of endovascular thrombectomy, as proposed in previous literature [13]. Focal and generalised slowing in EEG is highly unspecific, and it is unclear if even a full 10–20-resolution EEG would provide additional diagnostic value in acute stroke patients beyond detecting the unilateral disruption of the anterior circulation. Although the dense forehead array of the BrainStatus forehead electrode may not be ideal for detecting wide field slow activity, we believe the montage to be sufficient for our purpose. The four free channels on the amplifier already give this system some flexibility, e.g., to attach additional electrodes to extend the montage to more posterior regions of the brain. In the future we hope to have access to an electrode with fewer preset and more optional channels.

There are still issues to be solved. Most recordings suffered from some electrode–skin contact artefacts in at least one electrode and one recording was rendered completely unusable by insufficient electrode adhesion. Thus, EMS personnel must be carefully instructed in the application of the electrode set, as a loose reference electrode will almost certainly ruin the entire recording. The application technique should emphasise the importance of securing the contact of the reference electrodes above everything else.

This study provided evidence of a method of recording and wirelessly transferring good-quality EEG during an ambulance transfer. The next phase of this study will take the BrainStatus recorder to a prehospital setting by recording the EEG of 50 suspected acute stroke and brain insult patients during their transfer to Kuopio University Hospital’s emergency department and for up to two days during their stay in the hospital. Importantly, the first large-scale prehospital EEG study (ELECTRA-STROKE) [13] has shown that applying computational methods (e.g., theta/alpha-ratio) can improve the diagnostic accuracy of EEG in LVO-a. Our aim is to identify computational methods that can help detect patients with LVO-a from the recorded EEG also in real time. All the tools that will be used and the methods that will be evaluated will be released openly for anyone to utilise.

## Conclusions

Bittium BrainStatus, a CE-marked and commercially available compact EEG amplifier-recorder, is technically suitable for use in the ambulance. In our feasibility study the quality of the EEG signal was high and, at best, was sufficient for clinical decision-making. The wireless transfer of the EEG data to our remote server worked reliably. Some electrode contacts loosened during the transport, but this can likely be avoided with careful application of the electrode set. This system could be used to assess acute brain insult patients in the prehospital setting.
